# Visual Acuity and Size of Choroidal Neovascularization in Highly Myopic Eyes with a Dome-Shaped Macula

**DOI:** 10.1155/2020/8852156

**Published:** 2020-11-23

**Authors:** Lu Wang, Bin-wu Lin, Xiao-fang Yin, Wei-lan Huang, Yi-zhi Wang, Long Pang

**Affiliations:** ^1^Department of Ophthalmology, Guangdong Provincial Hospital of Chinese Medicine, Guangzhou 510000, China; ^2^Department of Ophthalmology, Foshan Hospital Affiliated to Southern Medical University, The Second People's Hospital of Foshan, Foshan 528000, China; ^3^Physical Examination Center, The Third Affiliated Hospital of Sun Yat-Sen University, Guangzhou 510000, China; ^4^Guangzhou University of Chinese Medicine, Guangzhou 510405, China

## Abstract

**Introduction:**

A dome-shaped macula (DSM) is an inward convexity or anterior deviation of the macular area. DSM is believed as a protective factor in maintaining visual acuity in highly myopic eyes.

**Objective:**

To investigate the correlation between best-corrected visual acuity (BCVA), choroidal neovascularization (CNV), and a dome-shaped macula (DSM) in highly myopic eyes.

**Methods:**

In this retrospective and observational case series study, BCVA tests and optical coherence tomography (OCT) were performed in a total of 472 highly myopic eyes (refractive error ≥6.5 diopters or axial length ≥26.5 mm). CNV was detected by fundus fluorescein angiography (FFA), and the CNV area was measured by ImageJ software. BCVA, central retinal thickness (CRT), and the CNV area were compared between highly myopic eyes with and without DSM.

**Results:**

The data revealed 13 eyes with DSM complicated by CNV, for an estimated prevalence of 25%. The eyes with CNV in the DSM group showed worse BCVA than those in the non-DSM group (1.59 ± 0.69 and 0.63 ± 0.64, respectively, *p* < 0.05), and the CNV area in the DSM group was larger than that in the non-DSM group (2793.91 ± 2181.24 and 1250.71 ± 1210.36 pixels, respectively, *p* < 0.05). After excluding the eyes with CNV, the DSM group had better BCVA than the non-DSM group (0.33 ± 0.17 and 0.44 ± 0.48, respectively, *p* < 0.05); however, no significant difference was observed in the CRT of eyes with CNV between the DSM group and the non-DSM group.

**Conclusion:**

These results show that DSM might be a protective mechanism for visual acuity, but its protective capability is limited. DSM eyes have better visual acuity within the protective capability. If a more powerful pathogenic factor exceeding the protective capability is present, then the eye will have more severe CNV and worse visual acuity.

## 1. Introduction

A dome-shaped macula (DSM) is an inward convexity or anterior deviation of the macular area that was originally defined by Gaucher et al. in 2008, who described DSM as a new type of myopic posterior staphyloma using optical coherence tomography (OCT) [1]. With the development of 3D MRI, other researchers found that DSM develops earlier than staphyloma formation in some patients. Recent studies have indicated that DSM can be found in emmetropic eyes and might be an independent factor for lesions [2, 3].

There are still many uncertainties regarding the pathophysiology of DSM configuration. Several theories have been published, such as scleral infolding, choroidal thickening, and vitreomacular traction, but none has been confirmed. Choroidal neovascularization (CNV), retinoschisis (RS), and serous retinal detachment (SRD) are the main complications that occur in DSM eyes [1, 4–9]. Among these complications, CNV was the most frequent, present in more than one-third, and it is a vital factor that threatens the visual acuity of highly myopic patients [8, 10]. Thinning of the sclera owing to long-term changes and elongation of the axis may develop CNV and other macular complications which could cause visual impairment in highly myopic eyes [11]. Prior reports have found an increase in the macular sclera and macular choroidal thickness in pathologic myopic eyes with DSM, and macular CNV was detected significantly more frequently in eyes without DSM than in eyes with DSM [12, 13]. It seems that greater bulge height and thicker choroid in highly myopic eyes with DSM may protect against the development of myopic CNV [13], given that DSM may serve as a compensatory mechanism. In addition, although myopic macular retinoschisis was detected more frequently in highly myopic eyes with DSM, the foveal retinoschisis was less common in eyes with DSM [9, 13]. Thus, it is believed that DSM was a protective factor in maintaining visual acuity in highly myopic eyes.

In this study, we evaluated the visual acuity of patients with highly myopic eyes with or without DSM. The features of CNV and the central retinal thickness (CRT) in patients with highly myopic eyes with CNV were also investigated and analyzed by fundus fluorescein angiography (FFA) and optical coherence tomography (OCT), respectively, which may provide evidence to further understand this phenomenon.

## 2. Materials and Methods

In this retrospective observational study, the medical records of patients with highly myopic eyes from the Guangdong Province Traditional Chinese Medical Hospital and The Second People's Hospital of Foshan were reviewed. This study adhered to the principles of the Declaration of Helsinki. High myopia was defined as a refractive error ≥−6.5 *D* or an axial length of >26.5 mm. Eyes with an inferior staphyloma due to congenital tilted disc syndrome or with other vision-threatening pathologies such as corneal opacity, severe cataracts, age-related macular degeneration, and diabetic retinopathy were excluded from the study. DSM was defined as an inward bulge of the macular RPE >50 *µ*m in the horizontal or vertical section of the OCT image [1].

All patients underwent a full ophthalmic examination including a best-corrected visual acuity (BCVA) test, tonometry, optometry, slip lamp examination, funduscopy, axial length (AL) measurements (IOL Master 500, version 7.7, Carl Zeiss AG, Dublin, California, USA), and OCT (3D OCT-2000, Topcon, Tokyo, Japan; Spectralis HRA + OCT, Heidelberg Engineering Inc., Germany). Vertical and horizontal line scans 6 mm in length and centered on the fovea were obtained from OCT. The height of the macular bulge compared with the bottom of the staphyloma was measured by tracing a line tangent to the outer border of the RPE at the bottom of the staphyloma. Then, the distance between the RPE at the foveal center and the newly traced line was measured, representing the height of the bulge.

Macular thickness was measured according to the Early Treatment of Diabetic Retinopathy Study (ETDRS) areas, which were defined by three concentric rings (central, inner, and outer circles) centered on the fovea, with diameters of 1, 3, and 6 mm. Other macular changes on OCT, such as intraretinal fluid (IRF), subretinal fluid (SRF), subretinal hyperreflective material (SHRM), myopic foveoschisis (MFS), the integrity of ellipsoid zone (EZ), and the type of CNV, were also recorded. Subjects with suspected CNV underwent FFA (TRC-50DX, Topcon, Tokyo, Japan; Spectralis HRA + OCT, Heidelberg Engineering Inc., German). The size of the CNV area was measured on FFA; areas with early hyper fluorescence and leakage were considered areas of CNV. All image processing and analyses were carried out using public domain software (ImageJ, v1.41d, available at http://rsb.info.nih.gov/ij). A single experienced examiner blinded to the clinical diagnoses of the subjects performed the OCT and FFA examinations.

### 2.1. Statistical Analysis

All statistical analyses were performed with SPSS for Windows software (version 17.0, SPSS, Inc., Chicago, IL). All values are expressed as mean ± standard deviation. Pearson's chi-square tests were used to compare categorical variables. Student's *t*-test was used to explore differences in means among continuous variables, and the Mann–Whitney test was performed when the sample data were not normally distributed. Therefore, the age, BCVA, size of CNV, and CRT were compared between the 2 groups using Student's *t*-test. The incidence of DSM in both sexes was compared between groups using chi-square tests. The IRF, SRF, SHRM, FS, type of CNV, and integrity of EZ were compared between groups using chi-square tests. A *p* value < 0.05 was considered statistically significant.

## 3. Results

### 3.1. Characteristics of Highly Myopic Eyes with DSM

**Figure 1:**
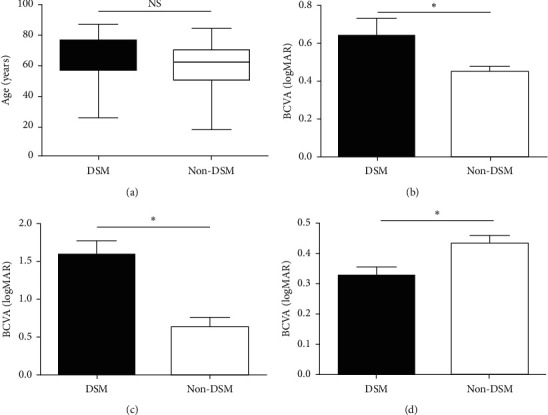
Average age and logMAR BCVA in patients with or without DSM. There is no statistical difference in the average age in DSM and non-DSM patients (a). The logMAR BCVA is significantly higher in patients with DSM compared to the patients without DSM (b). In patients with CNV, the logMAR BCVA is much higher in patients with DSM than non-DSM patients (c). In patients with no CNV lesions, the logMAR BCVA is much lower in patients with DSM than in patients without DSM (d).

A total of 472 highly myopic eyes of 366 patients from the Guangdong Province Traditional Chinese Medical Hospital and The Second People's Hospital of Foshan were identified. The database from October 2016 to April 2018 was included. Of the 472 eyes, 52 eyes (11.02%) of 42 patients had DSM (28/186 and 14/180, *χ*^2^ = 4.77, *p* < 0.05, Table 1). The average ages of patients with and without DSM were 64.7 ± 15.43 and 59.62 ± 15.21, respectively (*t* = 2.28, *p* > 0.05, Figure 1(a)). The other details are shown in Table 1.

Of the 472 myopic eyes, 40 eyes harbored CNV (8.5%): 13 eyes with DSM and 27 eyes without DSM.

The BCVA of myopic eyes with and without DSM was 0.64 ± 0.66 and 0.44 ± 0.50, respectively, which indicates that the DSM group had overall worse visual acuity than the non-DSM group (*t*′ = 2.2, *p* < 0.05, Table 1 and Figure 1(b)). Considering that CNV is a crucial complication that influences visual acuity, we further divided these 472 eyes into four subgroups: DSM eyes with CNV (13 eyes), non-DSM eyes with CNV (27 eyes), DSM eyes without CNV (39 eyes), and non-DSM eyes without CNV (393 eyes). The average BCVA in the four groups was 1.59 ± 0.69, 0.63 ± 0.64, 0.33 ± 0.17, and 0.44 ± 0.48, respectively (Table 1). The results showed that DSM eyes with CNV had poorer visual acuity than non-DSM eyes with CNV (*t* = 4.23, *p* < 0.05, Table 1 and Figure 1(c)). Once the eyes with CNV were excluded, the BCVA was better in eyes with DSM than in eyes without DSM (*t*′ = −2.96, *p* < 0.05, Table 1 and Figure 1(d)).

### 3.2. Characteristics of Highly Myopic Eyes with CNV

We further analyzed 40 highly myopic eyes that had been diagnosed with CNV by OCT and FFA. Of all 40 eyes, 13 highly myopic eyes had DSM, and 27 eyes did not have DSM. The average age was not significantly different between the two groups (65.33 ± 17.53 and 64.28 ± 15.73, respectively, *p* > 0.05, Table 2). The general characteristics of the highly myopic eyes associated with CNV are presented in Table 2. Notably, BCVA was significantly worse in DSM eyes with CNV compared to non-DSM eyes with CNV (1.59 ± 0.69 and 0.63 ± 0.64, respectively, *t*′ = −2.96, *p* < 0.05, Table 1 and Figure 1(c)).

### 3.3. The Size of CNV and Central Retinal Thickness (CRT) in Highly Myopic Eyes

**Figure 2:**
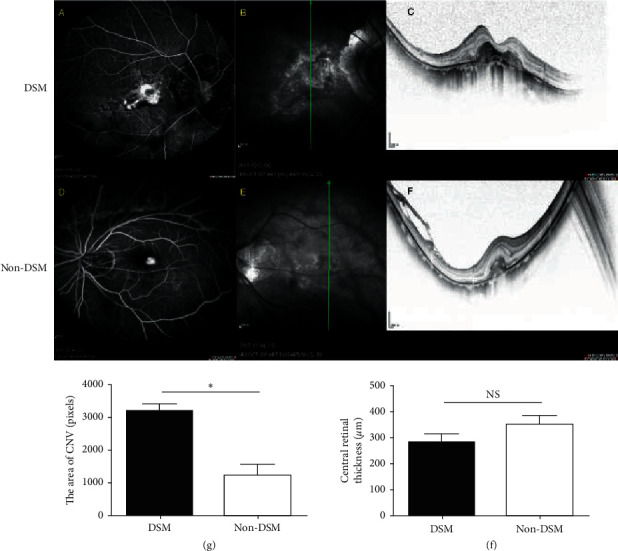
The area of CNV and the CRT in patients with CNV lesions. The pictures show typical FFA (a, d) and OCT (b, c, e, f) images in the patients with or without DSM. The area of CNV was dramatically higher in DSM patients than in non-DSM patients. No statistical difference has been found in central retinal thickness between the two groups.

The size of the CNV lesion and mean CRT were measured in 40 myopic eyes that had been diagnosed by OCT and FFA. The sizes of the selected CNV areas were 2793.91 ± 2181.24 pixels in the DSM group and 1250.71 ± 1210.36 pixels in the non-DSM group (*t*′ = 2.18, *p* < 0.05, Table 2 and Figure 2(g)). The CRTs were 284.25 ± 115.34 *μ*m in the DSM group and 353.35 ± 184.7 *μ*m in the non-DSM group (*t* = −1.197, *p* > 0.05, Table 2 and Figure 2(h)). These results indicated that the DSM group had larger CNV lesions than the non-DSM group; however, other potential factors affecting visual acuity, such as the average CRT, IRF, SRF, SHRM, FS, integrity of the EZ, and type of CNV, were not significantly different between the two groups (Table 2).

## 4. Discussion

In our study, DSM was observed in 52 of the 472 highly myopic eyes (11.02%), which is a similar rate to those reported by Ohsugi et al. (9.3%), Gaucher et al. (10.7%), Chebil et al. (12.0%), and I-Chia et al. (20.1%) [1, 8, 11, 14–16]. The present research showed that the DSM rate varies from 9.3% to 20.1%. The differences may be due to variations in the number of samples, differences in the inclusion criteria, and differences in the race of patients included in these studies.

The association between DSM and visual acuity remains controversial [9, 14, 17, 18]. DSM was initially believed to be a threat to visual acuity when first described by Gaucher et al. because of the association between DSM and several maculopathies, such as CNV, RS, SRD, foveal detachment, and RPE atrophy [1]. The incidence of maculopathy in DSM eyes is much higher than that in eyes without DSM [9, 19, 20].

This view was further confirmed by subsequent studies that focused on the morphological features of DSM, which found that DSM results from the relative thickening of the macular sclera and may lead to pigment epithelial detachment (PED). Long-term changes and elongation of the axis may thin the sclera, which leads to the development of CNV and causes visual impairment [11]; however, a larger-scale study suggested a different point. In a study involving 1118 highly myopic eyes, macular complications were more common in DSM eyes than in non-DSM eyes, and the most common complication was extrafoveal RS [9]. Foveal RS and CNV, two of the main threats to visual acuity, are much less common in DSM eyes than in non-DSM eyes [9, 10, 21]. Another study involving 1384 highly myopic eyes revealed that the incidence of foveal RS was also much lower in eyes with DSM than in those without DSM [16]. These results indicate that DSM might be a protective mechanism for visual acuity.

In this study, we found that the BCVA of patients in the non-DSM group was better compared to the DSM group. This result was confusing in view of the protective nature of DSM for visual function in highly myopic eyes. Given this situation, we compared the factors that may impair visual acuity including CRT, IRF, SRF, SHRM, FS, integrity of the EZ, type of CNV, and size of the CNV area between the two groups. The results showed that the CRT, IRF, SRF, SHRM, integrity of the EZ, and type of CNV were not significantly different between the two groups. The highly myopic eyes with DSM showed a larger CNV size than highly myopic eyes without DSM. Further analysis confirmed that the eyes with CNV in the DSM group showed worse BCVA than those in the non-DSM group. We hypothesize that patients in the DSM group had worse BCVA than patients in the non-DSM group due to the lower BCVA of eyes with CNV in the DSM than of eyes with CNV in the non-DSM group. After excluding eyes with CNV, the BCVA was better in the DSM group than in the non-DSM group. Additionally, as shown in the FFA results presented in Figure 2, the CNV area was larger in patients with CNV and DSM, but leakage and staining were also apparent. In patients with CNV without DSM, only leakage was observed. This result indicated the DSM patients have a longer duration of illness before asking a doctor for relief. In this case, we consider that DSM might be a protective mechanism for visual acuity, but its protective capability is limited. Less CNV and better visual acuity were found in DSM eyes within the protective capability. If a more powerful pathogenic factor exceeding the protective capability is present, then more severe CNV and worse visual acuity will occur. The development of CNV in DSM eyes indicates a powerful pathogenic factor, such as an elevated level of vascular endothelial growth factor (VEGF) and refractory macular diseases.

The precise cause of DSM remains unknown; however, several theories ranging from localized choroidal thickening to scleral infolding to vitreomacular traction have been postulated to underlie the mechanism and pathophysiological processes that lead to the formation of DSM in highly myopic eyes [22]. Imamura et al. showed that DSM resulted from the relatively localized thickening of the sclera under the macula in highly myopic eyes with enhanced depth imaging OCT [5]. The thick sclera in DSM eyes may act as a macular buckle-like mechanism, thereby alleviating tractional forces over the fovea, preventing foveal RS or RD (retinal detachment), and protecting visual function [9]. Such anatomical features may minimize refractive errors and maintain emmetropization in highly myopic eyes. Among the vision-threatening complications that can occur in highly myopic eyes, CNV is a key factor that can lead to severe visual impairment [23]. Different views exist about the relationship between the incidence of macular CNV and the presence of DSM. CNV has been reported to be a frequent complication of DSM (12.2%, 20.8%, 25.0%, 41.2%, and 47.8%) in previous studies [10, 24, 25]; however, Liang et al. reported that the incidence of macular CNV was associated with age but not the presence of DSM. The mechanism of CNV formation is unclear [8]. Akyol et al. considered that CNV development may be related to choroidal and retinal blood flow changes [26]. Ohsugi et al. reported that CNV formation is caused by thinning of the central sclera owing to elongation of the AL [11]. Other risk factors for the formation of CNV have been reported, such as lacquer cracks, choroidal thinning, patchy atrophy, and the presence of a choroidal filling delay [27, 28]. Thinning of the sclera owing to long-term changes and elongation of the axis may promote CNV development and cause visual impairment. Thus, in DSM eyes, a thick sclera may be a potential protective factor against CNV formation. The bulge height of DSM without the complication of CNV is significantly higher than that of DSM with the complication of CNV [11, 29].

In the current study, CRT, IRF, SRF, SHRM, FS, integrity of the EZ, and the type of CNV did not significantly differ between myopic eyes with and without DSM. In view of this observation, these factors are not the main cause of the visual acuity differences between eyes with CNV in the DSM group and those in the non-DSM group. The eyes with CNV in the DSM group had a larger CNV area than those in the non-DSM group. In a previous study, those with a larger CNV area developed more chorioretinal atrophy (CRA) than those with a smaller CNV area, which may explain why low BCVA was observed in myopic eyes with CNV and DSM [30]. As previously mentioned, the development of CNV in DSM eyes indicates the presence of a powerful pathogenic factor. The level of VEGF is the key factor in the formation of CNV. Therefore, the VEGF level may be higher in DSM eyes with CNV than in those without. This point of view was confirmed in a previous study that found that patients without DSM might be more sensitive to intravitreal ranibizumab therapy in early stages compared to patients with DSM [31].

Our study has several limitations. First, this retrospective study was subject to the potential inherent limitations associated with this study design. Second, we included patients who visited the Guangdong Province Traditional Chinese Medical Hospital and The Second People's Hospital of Foshan. Thus, the results obtained may not exactly reflect the general myopic population. Third, all patients were Chinese, and we did not include patients of other ethnicities. Fourth, patients with visual symptoms were more likely to be enrolled in the study, which might have resulted in a high incidence of CNV. Finally, no precise method to calculate the CNV size has been established. FFA and OCT are common methods and showed high consistency with previous research [32].

In summary, DSM is a progressive anomaly of the posterior pole of myopic eyes. Our study shows that DSM may play an important role in protecting highly myopic eyes complicated by severe maculopathy and maintaining good visual acuity initially; however, this protection is limited. When neovessels appeared, the size of the CNV area increased, and visual acuity worsened. This work may provide more evidence in predicting the visual prognosis of patients with highly myopic eyes and may provide additional insight into the mechanism of pathologic myopia. These conclusions indicated that the highly myopic patients with DSM need a more frequent follow-up to detect CNV in time. The DSM eyes with CNV might need a higher dose or more frequent intravitreal ranibizumab therapy in early stages than patients without DSM.

## Figures and Tables

**Table 1:** Characteristics of highly myopic eyes with and without DSM.

	DSM		*p* value
	Present (*n* = 52)	Absent (*n* = 420)	
No. of eyes (patients)	52 (45)	420 (321)	
Age (years)	64.7 ± 15.43	59.62 ± 15.21	>0.05
Sex (male/female)	14/28	166/158	<0.05
BVCA (logMAR)			
Total	0.64 ± 0.66	0.44 ± 0.50	<0.05
HM without CNV	1.59 ± 0.69	0.63 ± 0.64	<0.05
HM with CNV	0.33 ± 0.17	0.44 ± 0.48	<0.05

BVCA, best-corrected visual acuity; HM, highly myopic eyes.

**Table 2:** Characteristics of highly myopic eyes with CNV.

	DSM		*p* value
	Present (*n* = 13)	Absent (*n* = 27)	
No. of eyes (patients)	13(12)	27(25)	—
Age (years)	65.33 ± 17.53	64.28 ± 15.73	>0.05
Sex (male/female)	4/8	6/19	>0.05
The area of CNV (pixels)	2793.91 ± 2181.24	1250.71 ± 1210.36	<0.05
The CRT (*μ*m)	284.25 ± 115.34	353.35 ± 184.7	>0.05
The IRF (Y/N)	1/12	5/22	>0.05
The SRF (Y/N)	4/9	13/14	>0.05
The SHRM (Y/N)	3/10	8/19	>0.05
The type of CNV (1/2)	2/11	2/25	>0.05
The integrity of EZ (Y/N)	1/12	3/24	>0.05

BVCA: best-corrected visual acuity; HM: highly myopic eyes; CRT: central retinal thickness; CNV: choroidal neovascularization; IRF: intraretinal fluid; SRF: subretinal fluid; SHRM: subretinal hyperreflective material; EZ: ellipsoid zone.

## Data Availability

The data used to support the findings of this study are included within the article.
